# Nuclear Receptor SHP Activates miR-206 Expression via a Cascade Dual Inhibitory Mechanism

**DOI:** 10.1371/journal.pone.0006880

**Published:** 2009-09-01

**Authors:** Guisheng Song, Li Wang

**Affiliations:** Departments of Medicine and Oncological Sciences, Huntsman Cancer Institute, University of Utah School of Medicine, Salt Lake City, Utah, United States of America; Fondazione Telethon, Italy

## Abstract

MicroRNAs play a critical role in many essential cellular functions in the mammalian species. However, limited information is available regarding the regulation of miRNAs gene transcription. Microarray profiling and real-time PCR analysis revealed a marked down-regulation of miR-206 in nuclear receptor *SHP^−/−^* mice. To understand the regulatory function of SHP with regard to miR-206 gene expression, we determined the putative transcriptional initiation site of miR-206 and also its full length primary transcript using a database mining approach and RACE. We identified the transcription factor AP1 binding sites on the miR-206 promoter and further showed that AP1 (c-Jun and c-Fos) induced miR-206 promoter transactivity and expression which was repressed by YY1. ChIP analysis confirmed the physical association of AP1 (c-Jun) and YY1 with the endogenous miR-206 promoter. In addition, we also identified nuclear receptor ERRγ (NR3B3) binding site on the YY1 promoter and showed that YY1 promoter was transactivated by ERRγ, which was inhibited by SHP (NROB2). ChIP analysis confirmed the ERRγ binding to the YY1 promoter. Forced expression of SHP and AP1 induced miR-206 expression while overexpression of ERRγ and YY1 reduced its expression. The effects of AP1, ERRγ, and YY1 on miR-206 expression were reversed by siRNA knockdown of each gene, respectively. Thus, we propose a novel cascade “dual inhibitory” mechanism governing miR-206 gene transcription by SHP: SHP inhibition of ERRγ led to decreased YY1 expression and the de-repression of YY1 on AP1 activity, ultimately leading to the activation of miR-206. This is the first report to elucidate a cascade regulatory mechanism governing miRNAs gene transcription.

## Introduction

Small heterodimer partner (*SHP*, NROB2) is a well established nuclear transcriptional co-repressor. SHP interacts with a broad range of nuclear receptors and transcription factors and inhibits their transactivation [1]. In the past years, the metabolic regulatory function of SHP has been characterized using *SHP^−/−^* mice [2]–[4]. These studies revealed a diverse role of SHP in several metabolic diseases. Our recent study suggests a new aspect of SHP regulation in the development of hepatocellular carcinoma (HCC), which is associated with SHP inhibition of cellular proliferation and activation of apoptosis signaling [5], [6].

MicroRNAs (miRNAs, miR) are highly conserved small RNA molecules of 22 nucleotides in length which regulate the gene expression by binding to the 3′-untranslated regions (3′-UTR) of specific mRNAs [7]. Despite the growing evidence for their importance in development, proliferation, and differentiation [8]–[10], limited information is available about how miRNAs are regulated transcriptionally. To determine the regulation of SHP in miRNAs expression and function, we recently cloned two overlapping primary transcripts encoding miR-433 and miR-127, respectively [11]. The coupled miR-433 and miR-127 were transcribed from independent promoters repressed by SHP in a compact space by using overlapping genomic regions [12]. Our study identified SHP as an important transcriptional regulator of miRNAs gene expression.

In this study, we cloned the full length primary transcript of miR-206 and elucidated a regulatory cascade activating miR-206 expression by SHP which involved AP1 (transcription factor activator protein 1), YY1 (Ying Yang 1), and ERRγ (estrogen related receptor gamma). This is the first report to elucidate a cascade regulatory mechanism governing miRNAs gene transcription.

## Results

### Identifying decreased expression of miR-206 in *SHP^−/−^* mice and determining miR-206 full length primary transcript

**Figure 1:**
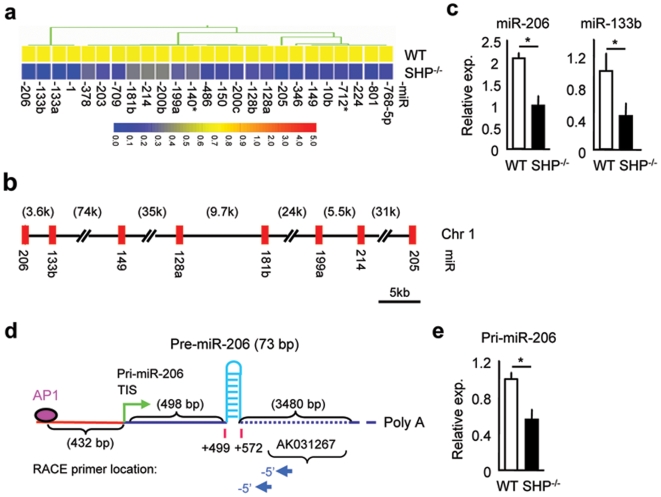
Cloning of full length pri-miR-206 in the livers of *SHP^−/−^* mice. (a) Hierarchical clustering of the down-regulated miRNAs in the livers of *SHP^−/−^* mice compared to wild-type (WT) mice. (b) Schematic of the chromosomal location of the down-regulated miRNAs in *SHP^−/−^* mice on chromosome 1. (c) Real-time PCR verification of the miR-206 and miR-133b expression in the livers of WT and *SHP^−/−^* mice. (d) Schematic of the genomic structure of miR-206 gene. The gene for miR-206 is located on chromosome 1 on the positive strand. The green arrow represents the putative transcriptional initiation site (TIS) of pri-miR-206, which is 498 bp upstream of pre-miR-206. The navy arrows indicate the location of 5′RACE primers used to identify TIS. TFs, transcription factor sites. (e) Real-time PCR analysis of pri-miR-206 expression in the livers of WT and *SHP^−/−^* mice. Primers are located within the 5′RACE amplified region. Data in c and e are represented as mean±SEM. *Significantly different (p<0.01).

A custom microarray identified a subset of miRNAs that were differentially down-regulated in livers of *SHP^−/−^* mice, which exhibited a 2-fold or greater decrease in expression (Figure 1a). Two clusters of miRNAs, miR-206/miR-133b on chromosome 1 and miR-1/miR-133a on chromosome 2 showed the largest magnitude of down-regulation (Table S1). Interestingly, a cluster of other down-regulated miRNAs was observed on chromosome 1 (Table S2), which were more distantly located (Figure 1b). Real-time PCR analysis confirmed that the expression level of miR-206 and miR-133b was decreased by an average of 50% to 60% in the liver of *SHP^−/−^* mice than in wild-type (WT) controls (Figure 1c). Interestingly, the basal expression of miR-206 was about 2-fold higher than miR-133b, suggesting that the paired miR-206 and miR-133b might be derived from two primary transcripts under the control of independent promoters, similar to the paired miR-433 and miR-127 [11], [12]. It was noted that the extent of miR-206 down-regulation was higher by microarray than by real-time PCR. However, real-time PCR was generally considered as a more quantitative method for gene expression analysis. Nevertheless, both methods produced similar expression profiles for miR-206 and showed decreased expression of miR-206 in *SHP^−/−^* mice. The down-regulation of these miRNAs in *SHP^−/−^* mice suggested that they were potential transcriptional targets of SHP.

**Figure 2:**
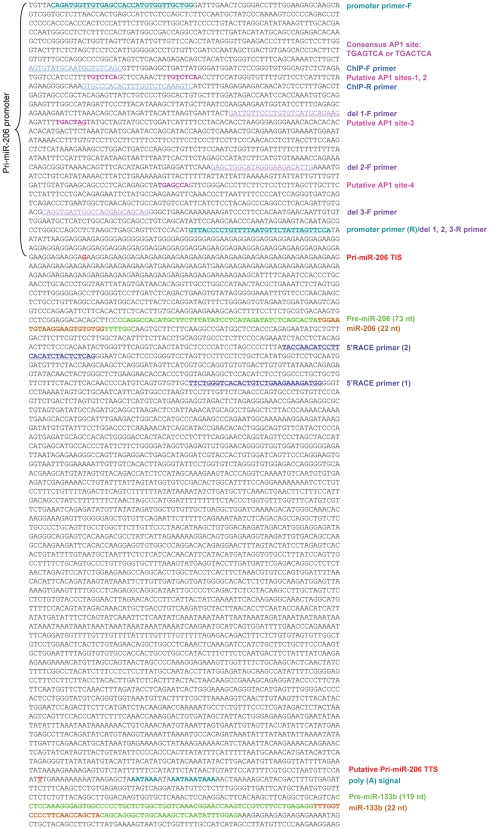
Genomic sequences of the miR-206 gene, including the full length sequences of the pri-miR-206 primary transcript and the miR-206 promoter region. Putative AP1 binding sites are indicated (pink). Primer sequences for RACE (purple), promoter cloning (aqua), promoter deletion construct (magenta), and ChIP assays (blue): underlined; TIS, transcriptional initiation site; TTS, transcriptional termination site. The color in word description matches with the color of the gene sequences.

We focused on determining the primary transcript encoding miR-206 because the basal level of miR-206 in the liver was much higher than miR-133b (Table S1). For this we used a bioinformatics approach developed in our laboratory [11]. Expressed sequence tag (EST) and non-coding RNA database (mouse non-RefSeq RNA database from NCBI) analysis identified an EST sequence (AK031267, GenBank Accession number) ended before pre-miR-133b (Figure 2–2, T in red), which was followed by the consensus polyadenylation signal. This suggested that the 3′-end of this EST was complete. The 5′-end of this EST ended close to the 3′-end of the miR-206 hairpin sequences and did not contain miR-206, based on the genomic location of pre-miR-206 (Figure 1d). This suggested that it did not contain the full length pri-miR-206. Sequence prediction suggested that miR-206 and miR-133b may arise from two separate, and possibly overlapping primary transcripts. This prediction is consistent with the report that pri-miRNA transcripts vary in length from a few hundreds of bases up to tens of kilobases [13]. To elucidate the transcriptional initiation site (TIS) of the primary transcript of miR-206 (pri-miR-206), we used Rapid Amplification of cDNA Ends (RACE) to determine the 5′-end of the transcript. 5′RACE produced one strong and two weak PCR products (Figure S1a). However, a single TIS from the strongest PCR product was confirmed to be specific by sequencing analysis, which was located 498 nt upstream of pre-miR-206 (Figure 1d, Figure S1b, and Figure 2–1). The two weak bands appeared to be non-specific PCR amplification. In addition, the expression level of pri-miR-206, as detected using primers in the 5′RACE amplified region, was about 50% lower in *SHP^−/−^* mice as compared to the wild-type (WT) mice (Figure 1e), consistent with the expression pattern of the mature miR-206 (Figure 1c). Because this TIS site has not been verified using additional experimental approaches such as primer extension assays, we considered this as a putative TIS site. The identification of this putative TIS allowed us to determine the location of the miR-206 promoter and to clone it for transcriptional analysis of miR-206 expression.

### YY1 inhibits miR-206 promoter transactivation by AP1

A number of studies have established SHP as a transcriptional repressor. Thus, the decreased miR-206 expression in *SHP^−/−^* mice indicated a secondary effect due to the loss of SHP repression. We hypothesized a “dual inhibitory mechanism”, by which SHP repressed an intermediate gene that inhibited the miR-206 promoter resulting in a final outcome of SHP activating pri-miR-206 expression.

The transcription factor activator protein 1 (AP1) is a heterodimer nuclear protein composed of the proto-oncogene products c-Jun and c-Fos, and is involved in regulation of cell proliferation and tumor promotion [14]. AP1 can activate its target gene promoters through AP1 binding elements. Yin Yang 1 (YY1) is a multifunctional protein that plays a fundamental role in development, differentiation, replication, and cellular proliferation [15]. YY1 exerts its effects via its ability to initiate, activate, or repress transcription depending upon the context of the cells and promoters. Sequence analysis of the miR-206 promoter with the MatInspector program predicted four potential binding sites for AP1 (Figure 2–1, pink). Because it has been reported that YY1 can inhibit c-Jun activity by direct protein-protein interaction [16], we investigated whether YY1 could suppress AP1 activity in the miR-206 promoter.

**Figure 3:**
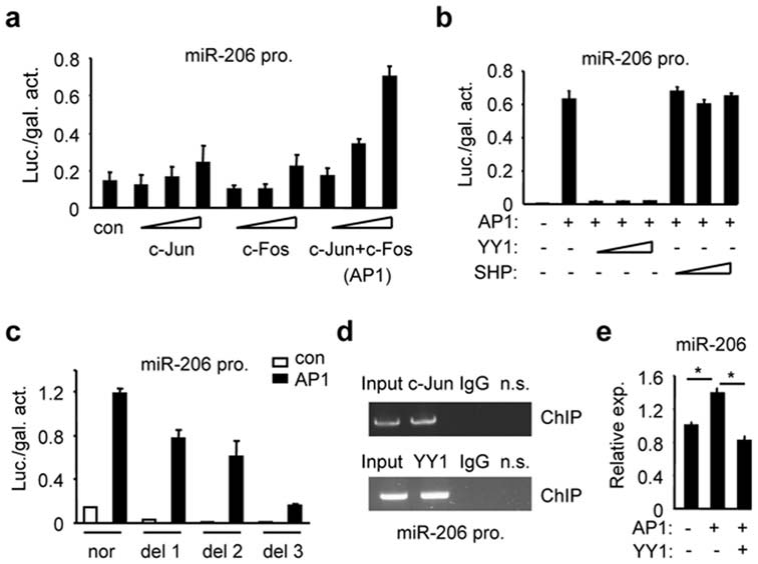
YY1 inhibits AP1 activation of the miR-206 promoter and expression. (a) Transient transfection assays to determine AP1 (c-Jun/c-Fos, 50, 100, 200 ng) transactivation of pri-miR-206 promoter (pro.). The promoter of pri-miR-206 was cloned into a pGL3-basic vector. Hela cells were transfected with the miR-206Luc in the presence of c-Jun and/or c-Fos plasmids. Luciferase (luc.) activities (act.) were determined, which were normalized by β-gal (gal.) activities. Con, control (pcDNA3). (b) YY1 (50, 100, 200 ng) inhibition of miR-206Luc activity by AP1 (200 ng). (c) AP1 (200 ng) activation of miR-206Luc deletion constructs (nor, normal promoter containing 4 putative AP1 sites; del 1, AP1 sites 1 and 2 deleted; del 2, AP1 site 3 deleted; del 3: AP1 site 4 deleted). Con, control (pcDNA3). (d) ChIP assays of c-Jun and YY1 Co-immunoprecipitation (Co-IP) on the miR-206 promoter region containing putative AP1 sites 1 and 2. (e) Real-time PCR analysis of miR-206 expression in Nmuli cells with AP1 and YY1 overexpression. Data in a, b, c, and e are represented as mean±SEM. *Significantly different (p<0.01).

Overexpression of c-Jun or c-Fos alone showed marginal activation of the miR-206 promoter, while overexpression of AP1 containing c-Jun and c-Fos significantly transactivated the miR-206 promoter in a dose-dependent manner (Figure 3a). However, this positive regulation was abrogated by YY1 co-expression (Figure 3b). In contrast, no effect was observed with SHP alone (not shown) or co-expression on AP1 activity.

Mutagenesis studies by mutating the upstream two AP1 sites (sites 1&2) in the miR-206 promoter decreased AP1 activity, but did not abolish it (Figure S2), suggesting that other putative AP1 sites may also contribute to the AP1 responsiveness. Therefore, three miR-206 promoter luciferase (pro. Luc) reporter deletion constructs were generated in which putative AP1 sites were sequentially deleted (Figure 2–1, see primer locations used for deletion constructs). Transient transfection assays showed that deletion of AP1 sites 1 and 2 (del 1 pro. Luc) decreased AP1 activity as compared to the normal miR-206 pro. Luc (Figure 3c). Deletion of AP1 site 3 (del 2 pro. Luc) did not further decrease AP1 activity, whereas deletion of AP1 site 4 (del 3 pro. Luc) significantly diminished AP1 activity. Thus, sites 1, 2 and 4 in the miR-206 promoter appeared to be potent AP1 sites for AP1 activation.

Chromatin immunoprecipitation (ChIP) analysis with a primer set covering the AP1 sites 1 and 2 confirmed the physical association of AP1 and YY1 with the endogenous miR-206 promoter in mouse hepatoma Hepa-1 cells by using specific c-Jun and YY1 antibodies (Figure 3d). In contrast, a non-specific (n.s.) primer set located ∼11 kb downstream of miR-206 promoter did not produce PCR product. Nevertheless, overexpression of AP1 (c-Jun & c-Fos) induced miR-206 expression which was decreased by YY1 co-expression (Figure 3e). To this point, we conclude that the miR-206 promoter can be potently transactivated by AP1 and this response can be reversed by YY1.

### SHP inhibits YY1 promoter transactivation by ERRγ

**Figure 4:**
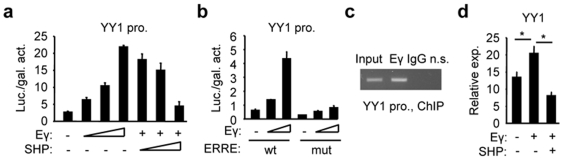
SHP inhibits ERRγ activation of YY1 promoter and expression. (a) SHP repression of YY1 promoter transactivation by ERRγ. Transient transfection assays to determine ERRγ (20, 40, 80 ng) transactivation and SHP (100, 200, 400 ng) transrepression of YY1 promoter (pro.). (b) Mutagenesis assays. The ERRE of the YY1 promoter Luc was mutated and used for transient transfection. (c) ChIP assays of ERRγ Co-immunoprecipitation (Co-IP) on the YY1 promoter region containing a putative ERRE. (d) Real-time PCR analysis of YY1 mRNA expression in Nmuli cells with ERRγ and SHP overexpression. Data in a, b, and d are represented as mean±SEM. *Significantly different (p<0.01).

We hypothesized that YY1 might be the intermediate gene through which SHP regulated miR-206 expression. Although the YY1 promoter had more than 90% GC content and was initially difficult to amplify, we successfully cloned it into a luciferase reporter (Figure S3a). ERRγ is a nuclear receptor and a target for SHP repression [3], [12]. A conserved estrogen related receptor response element (ERRE) was identified in the YY1 promoter (Figure S3b). As predicted, ERRγ dramatically activated YY1 promoter, which was repressed by expression of SHP (Figure 4a). Mutation of the ERRE in the YY1 promoter reduced ERRγ activity below the basal level (Figure 4b). ChIP analysis using specific ERRγ antibodies confirmed ERRγ binding to the YY1 promoter in Hepa-1 cells (Figure 4c), in which ERRγ showed increased expression compared to normal mouse hepatocye Nmuli cells [12]. Finally, the expression of YY1 mRNA was increased by ERRγ and decreased by SHP (Figure 4d). The data identified ERRγ and SHP as novel transcriptional regulators of YY1 gene expression.

### Cascade transcriptional activation of miR-206 by SHP

**Figure 5:**
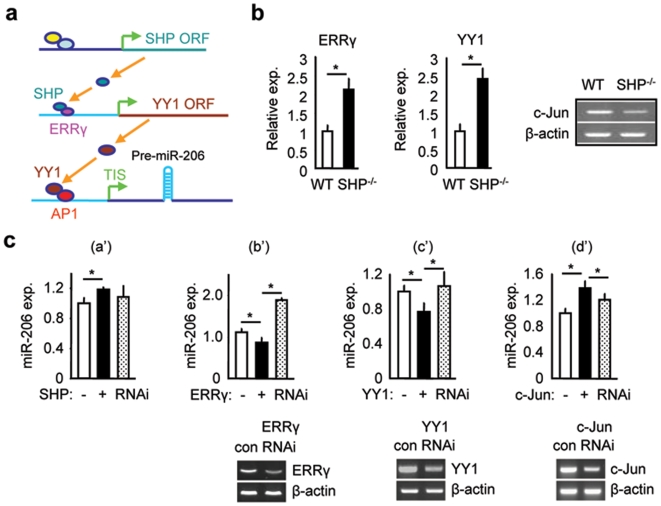
A “dual-inhibitory” mechanism activating miR-206 gene transcription by SHP. (a) Proposed cascade regulatory model activating miR-206 expression by SHP. (b) Real-time PCR analysis of ERRγ and YY1 mRNA expression and semi-quantitative PCR analysis of c-Jun mRNA expression in the livers of *SHP^−/−^* and wild-type (WT) mice. (c) Top: real-time PCR analysis of miR-206 expression in Nmuli cells with SHP (a'), ERRγ (b'), YY1 (c') and AP1 (d') overexpression or knockdown. Bottom: semi-quantitative PCR analysis of ERRγ (left), YY1 (middle), and c-Jun (right) expression level in cells transfected with a control (con) or gene specific shRNA against ERRγ, YY1, and c-Jun, respectively. Data in b and c are represented as mean±SEM. *Significantly different (p<0.01).

Based on the above experimental results, we propose a cascade regulatory model of miR-206 expression. In this model, SHP inhibits ERRγ activation of the YY1 promoter and YY1 represses AP1 activation of the miR-206 promoter. Thus, SHP inhibition of ERRγ leads to decreased YY1 expression and the de-repression of YY1 on AP1 activity, which ultimately leads to the increase in miR-206 expression (Figure 5a). In support of this model, the expression of YY1 and ERRγ was increased whereas the expression of c-Jun was decreased in livers of *SHP^−/−^* mice (Figure 5b), which corresponded to the down-regulation of miR-206 (Figure 1).

We further tested the effect of each individual nuclear receptor and transcription factor on miR-206 expression in Nmuli cells. As expected, SHP overexpression resulted in an induction of miR-206 expression (Figure 5c–a'). Unfortunately, we could not obtain satisfactory results with SHPRNAi due to the low SHP levels and knockdown efficiency in Nmuli cells. Expression of ERRγ caused a reduction of miR-206 levels, which was reversed in ERRγ-siRNA transfected cells (Figure 5c–b'). In a similar way, an inhibitory effect of miR-206 was observed in YY1 overexpressed cells and the repression was absent when YY1 levels were decreased by siRNA (Figure 5c–c'). Consistent with the previous results, AP1 (c-Jun & c-Fos) induced miR-206 expression and the effect was partially blocked by c-Jun knockdown (Figure 5c–d'). Considering together, these results demonstrate a cascade regulatory mechanism governing miR-206 gene transcription which involved SHP, ERRγ, YY1, and AP1.

## Discussion

MiR-206 was initially identified as a skeletal muscle specific miRNA [17] that played an important function in muscle development [18]–[20]. Recent studies showed that miR-206 was downregulated in estrogen receptor (ER) positive breast cancer [21], which may be associated with ER as a miR-206 target [22]. Thus, miR-206 is suggested to function as a suppressor of breast cancer metastasis [23], although the mechanism remains to be defined. In addition, the expression of miR-206 in the brain has been associated with schizophrenia [24]. Recently, specific expression of miR-206 was reported in brown adipocytes [25] and the expression level of miR-206 was also increased in bone marrow-derived DC19^+^ WM cells associated with Waldenstrom macroglobulinemia [26]. Although miR-206 is less abundant in the liver, our study identified down-regulation of miR-206 in the liver of *SHP^−/−^* mice. These observations suggest a broader tissue specific expression and physiological function of miR-206 than originally anticipated.

Despite the important function of miR-206 in physiological regulation, how the expression of miR-206 is controlled at the transcriptional level remains unknown. To address this question, we first cloned the full length pri-miR-206 using a bioinformatics approach. Cloning of pri-miR-206 is significant, because only a few miRNAs have their full length primary sequences determined [11], [27], [28]. It is noted that the identified transcriptional initiation site of pri-miR-206 is localized in a simple GGA/GAA sequence repeat region, with no identifiable core promoter elements. However, this feature is not unusual for the miRNA genes. Our previous studies identified (CT)n or (CTT)n simple sequence repeats in the promoter of the primary transcript of miR-127 [11], [12]. Another study also showed that TATA-box was not common for most miRNA genes in *C. elegans* and *H. sapiens*, although most studied miRNA genes of A. thaliana and O. sativa contained TATA-box [29]. In addition, several other studies reported that (CT)n, (CCT)n, (CTT)n, (CCTT)n, (CGCT)n, (CCTCG)n, (CCTCT)n, (CGTCT)n, and (CTCTT)n simple sequence repeats were the significant motifs in core promoters of miRNA Genes [30]–[33].

A major funding of this study is the identification of a cascade regulatory mechanism governing miR-206 expression by SHP. Genes encoding miRNAs are transcribed by RNA polymerase II [27] and in some cases RNA polymerase III [34]. However, little is known about how transcriptional regulation affects miRNAs levels and function in cells and tissues. Using both an *in vitro* cell system and *in vivo* gene expression analysis, we show that several nuclear transcription factors and signaling molecules, including SHP, ERRγ, YY1, and AP1, coordinately regulate the transcription of miR-206. Thus, SHP is identified as a transcriptional activator of miR-206 expression via a “dual inhibitory” mechanism. Because the expression of miR-206 is also markedly down-regulated in skeletal muscle of *SHP^−/−^* mice compared to the wild-type mice (Figure S4), where it is preferentially expressed, we propose that this transcriptional cascade that activates miR-206 by SHP may exist in muscle as well. The data suggests that complexity mRNA-miRNA interactions participate in miR-206 control of multiple cellular processes.

## Methods

### Total RNA isolation and miRNA microarray analysis

Protocols for animal use were approved by the Institutional Animal Care and Use Committee at the University of Utah. Total RNA with miRNA was isolated from the liver of two month old male mice (n = 3) using *mir*Vana™ miRNA Isolation Kit (Ambion, Austin, TX). The RNA quality control was performed using Bioanalyzer 2100. SHP knockouts (*SHP^−/−^*) and wild type mice on a pure C57/BL6 background were analyzed. The mice were given normal chow diet under feeding conditions. MiRNA microarray including labeling, hybridization, image scanning and initial data analysis was carried out by LC sciences (http://www.LCsciences.com, Houston, TX). All protocols were deposited at ArrayExpress. LC-miRHumanMouseRat-9.1-070207-MRA-1030 array was used which was deposited in MIAMExpress. In brief, arrays were made based on μParaflo microfluidic technology (Atactic Technologies). On the μParaflo microfluidic chip, each detection probe consisted of a chemically modified nucleotide coding segment complementary to target microRNA (from miRBase, http://microrna.sanger.ac.uk/sequences/) or other RNA (control or customer defined sequences) and a spacer segment of polyethylene glycol to extend the coding segment away from the substrate. The detection probes were made by *in situ* synthesis using photogenerated reagent (PGR) chemistry (Array Protocol: LC Mir-Array-Prtl-060518). Small RNAs (<300 nt) were 3′-extended with a poly(A) tail using poly(A) polymerase. An oligonucleotide tag was then ligated to the poly(A) tail for later fluorescent dye staining; two different tags were used for the two RNA samples in dual-sample experiments (Labeling Protocol: LC Mir-Label Prtl-060518). Hybridization was performed using a micro-circulation pump (Atactic Technologies). The hybridization conditions were 100 µL 6xSSPE buffer (0.90 M NaCl, 60 mM Na_2_HPO_4_, 6 mM EDTA, pH 6.8) containing 25% formamide, 34°C, and overnight (Hybridization Protocol: LC Mir-Hyb Prtl-060518). Hybridization images were collected using a laser scanner (GenePix 4000B, Molecular Device). Scan resolution was set at 10 µ and PTM is set between 350 to 700 V (Scanning Protocol: LC Mir-Scan Prtl-060518). Data were analyzed by first subtracting the background and then normalization. The background was determined using a regression-based background mapping method. The regression was performed on 5% to 25% of the lowest intensity data points excluding blank spots. Raw data matrix was then subtracted by the background matrix. Normalization was carried out using a LOWESS (Locally-weighted Regression) method on the background-subtracted data (Normalization Protocol: LC Mir-Norm Prtl-060518). The data was deposited to the ArrayExpress database and the accession number is E-MEXP-1721 [12].

### Database mining and EST extension

MiRNAs precursor sequences were downloaded from the Sanger Institute (http://microrna.sanger.ac.uk/ sequences). The BLASTN search of the mouse genome was done online. The mouse EST and ncRNA database were downloaded from GenBank. A 3 kb genomic sequence centered in the miRNA precursor was extracted manually and used as query to search the EST and ncRNA database with the command “Megablast -e 1e-100 -F “m L” -D 3”. The Blast Packages (v2.2.10) were downloaded from the NCBI website (ftp://ftp.ncbi.nlm.nih.gov.blast/executables). The ncRNA and ESTs with the opposite transcription direction compared to query were removed from the Blast hits. The hits with an aligned length less than 95 of their original length were filtered out. After filtering, the ESTs matched to the −2 kb to 2 kb flanking regions were selected for further analysis [11].

### Real-time RT-PCR quantification of miRNAs

Real-time reverse transcription polymerase chain reaction (RT-PCR) quantification of miRNA expression was carried out using TaqMan® MicroRNA Assays Kit (Applied Biosystems Inc. Foster City, CA) according to manufacturer's protocol. snoRNA202 was used as an internal control to normalize RNA input in the real-time RT-PCR assay. The detailed method was described in our recent publication [12].

### RACE mapping of miRNA primary transcript

Total liver RNA was isolated using an RNeasy Mini Kit (Qiagen, Valencia, CA) and mRNA was isolated using an Oligotex Direct mRNA Mini Kit (Qiagen, Valencia, CA). The GeneRacer Kit (Invitrogen, California, USA) was used to map the transcriptional initiation site of primary transcript. The first strand cDNA was synthesized at 65**°**C with Thermo-X™ reverse transcriptase using 2 µg of mRNA, followed by a polymerase chain reaction with 95**°**C denaturation step and then 45 cycles of touchdown annealing temperature. Primer sequences are indicated in Figure 2. The genomic sequences of the miR-206 gene was deposited in GenBank, Accession number FJ469647.

### Transient transfection and luciferase assay

Expression plasmids of SHP, ERRγ, YY1, c-Jun, and c-Fos were cloned into the pcDNA3 vector. Luciferase reporters of miR-206 and YY1 were cloned into the pGL3 reporter construct (Promega, Madison, WI). Twenty four hours before transfection, 5×10^4^ cells were plated per well in a 24-well plate. 30 ng of miR-206 or YY1 luciferase reporter construct, different concentrations of expression plasmid, and 30 ng of *beta-gal* plasmid pSV-β-Galactosidase Control Vector (Promega, Madison, WI) were transfected using FuGENE HD (Roche, Indianapolis, IN). Different amounts of expression vector pcDNA3 were added to keep the final amount of DNA constant for all transfections. Thirty six hours after transfection, luciferase and β-galactosidase assays were performed using the Luciferase Assay System system and Beta-Glo® Assay System (Promega, Madison, WI). Luciferase activities were normalized to galactosidase activities for each transfected well. For each experimental trial, wells were transfected in triplicate and each well was assayed in triplicate.

### Chromatin immunoprecipitation (ChIP) assays

ChIP Assays were performed using the ChIP Assay Kit (Upstate Biotechnology, Lake Placid, NY). Hepa-1 cells were cultured until 70%–80% confluence. Chromatin was cross-linked with 1% formaldehyde at 37**°**C for 10 min. Cells were washed with cold PBS twice and disrupted in SDS Lysis Buffer containing the protein inhibitor cocktail. Chromatin was sonicated to shear DNA to an average length between 200 bp and 1000 bp as verified by agarose gel. The sonicated cell supernatants were diluted 10 fold in ChIP Dilution Buffer containing the protein inhibitor cocktail and an aliquot of the solution was reserved for input control. Ten micrograms of YY1 (Abcam, Cambridge, MA), ERRγ (Aviva Systems Biology, San Diego, CA), or c-Jun (Abcam, Cambridge, MA) antibodies were added and the chromatin solution was gently rotated overnight on ice. The protein A agarose slurry was added to the antibody bound chromatin solution and incubated at 4°C for 1 hr with constant rotation. The agarose beads were collected by centrifugation, washed and the antibody bound chromatin was released from the agarose beads. Finally, the DNA was purified by phenol/chloroform extraction and ethanol precipitation. The binding region was detected using primers in PCR reactions. A 10 kb region downstream from the binding site was used as negative control (n.s.). All ChIP primers used for miR-206 promoter and YY1 promoter are indicated in Figure 2-1 and Figure S2b. The non-specific primers for miR-206 promoter ChIP assays are: F: 5′-CTACTTATGCAGCTAGAGATACAAG-3′ and R: 5′-ACTTCCAATAAGTCTTTGACCCATG-3′.

### RNA interference (RNAi)

ShRNA constructs against *Mus musculus* ERRγ, SHP, YY1 and c-Jun were purchased from Origene Company (Rockville, MD). Nmuli cells were cultured until 70%∼80% confluence. ShRNA constructs were transfected into the cells using transfection reagent Fugene HD (Roche) according to the manufacturer's instructions. The level of miR-206 expression was determined using real-time PCR.

### Statistical analysis

All the experiments were repeated at least three times and the error bars represent the standard error of the mean (SEM). Statistical analyses were carried out using Student's unpaired t test; p<0.01 was considered statistically significant.

## Supporting Information

Table S1 MiRNAs with the largest magnitude of down-regulation in SHP−/− mice. miR-206 and miR-133b were clustered on chromosome 1, whereas miR-1 and miR-133a were clustered on chromosome 2. The expression level of miR-206 was markedly higher than miR-133b, and the expression level of miR-1 was markedly higher than miR-133b.
(1.30 MB TIF)

Table S2 Down-regulated miRNAs in SHP−/− mice on chromosome 1.
(1.30 MB TIF)

Figure S1 (a) Determining the putative transcriptional initiation site (TIS) by 5′-RACE for pri-miR-206. Total liver RNA was isolated using RNeasy Mini Kit (Qiagen, Valencia CA) and mRNA was isolated using a Oligotex Direct mRNA Mini Kit (Qiagen, Valencia CA). A GeneRacer Kit (Invitrogen, California USA) was used to map the transcriptional initiation site of the primary transcript. (b) Chromatogram of 5′ RACE sequences of the pri-miR-206 primary transcript. The putative transcriptional initiation site of pri-miR-206 (G) is indicated by a pink arrow.
(3.89 MB TIF)

Figure S2 Mutagenesis assays. Two upstream putative AP1 sites (sites 1&2) (see Figure 2–1 for the location of each site) of the miR-206 promoter was mutated by using the QuickChange XL site-Directed Mutagenesis Kit (Stratagene), which generated the mutated miR-206 promoter luciferase reporter mut1 (single AP1 site 1 mutation) and mut1-2 (double AP1 sites 1&2 mutation). For luciferase reporter experiments, 30 ng of wt, mut1, mut1-2 and 30 ng of β-gal plasmid pSV-β-Galactosidase control vector were co-transfected with the AP1 expression vector (80 ng) into Hela cells using FuGENE HD (Roche). Thirty six hours after transfection, luciferase and β-galactosidase assays were performed using the Luciferase Assay System system and Beta-Glo® Assay System (Promega). Luciferase activities were normalized to galactosidase activities for each transfected well. For each experiment, wells were transfected in triplicate and each well was assayed in triplicate.
(3.89 MB TIF)

Figure S3 (a) Cloning of the high GC content mouse YY1 promoter. The PCR product was cloned into a pGL3-basic vector and used for transfection assays. The construct was verified by sequencing. (b) YY1 promoter sequences. TSS, transcriptional start site; ERRE, putative ERR binding site; YY1 pro. F and R, forward and reverse primers used to clone the YY1 promoter (pro.) and for ChIP assays.
(3.89 MB TIF)

Figure S4 Real-time PCR analysis of miR-206 expression in skeletal muscle of SHP−/− and SHP+/+ mice. Data is represented as mean±SEM. *Significantly different (p<0.01).
(3.89 MB TIF)
