# Supplementary figures and images for: Nuclear Receptor SHP Activates miR-206 Expression via a Cascade Dual Inhibitory Mechanism
Source: PLoS One. 2009 Sep 1;4(9):e6880. doi: 10.1371/journal.pone.0006880 (PMC2730526; doi:10.1371/journal.pone.0006880)

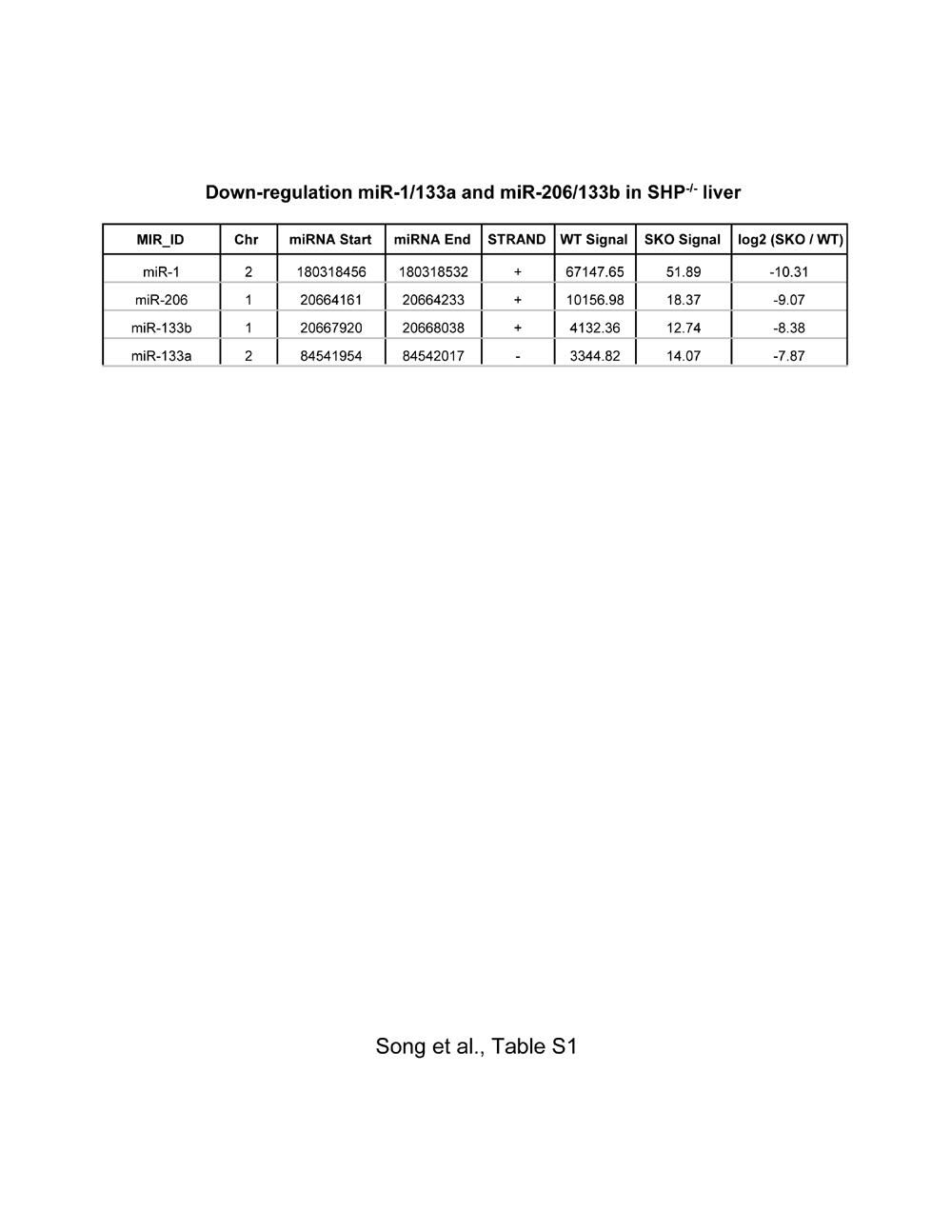

Supplement: Table S1 — MiRNAs with the largest magnitude of down-regulation in SHP−/− mice. miR-206 and miR-133b were clustered on chromosome 1, whereas miR-1 and miR-133a were clustered on chromosome 2. The expression level of miR-206 was markedly higher than miR-133b, and the expression level of miR-1 was markedly higher than miR-133b. (1.30 MB TIF) [file pone.0006880.s001.tif]

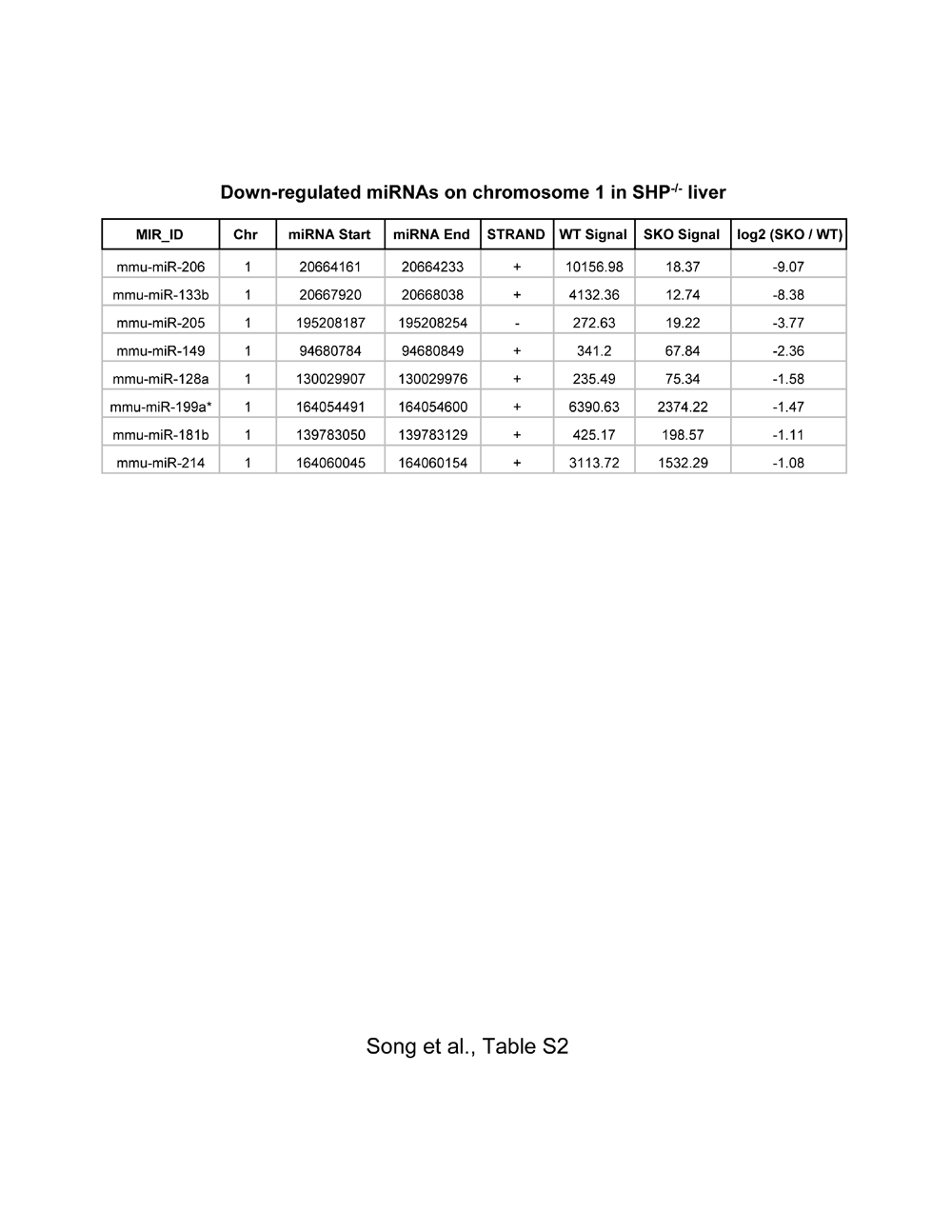

Supplement: Table S2 — Down-regulated miRNAs in SHP−/− mice on chromosome 1. (1.30 MB TIF) [file pone.0006880.s002.tif]

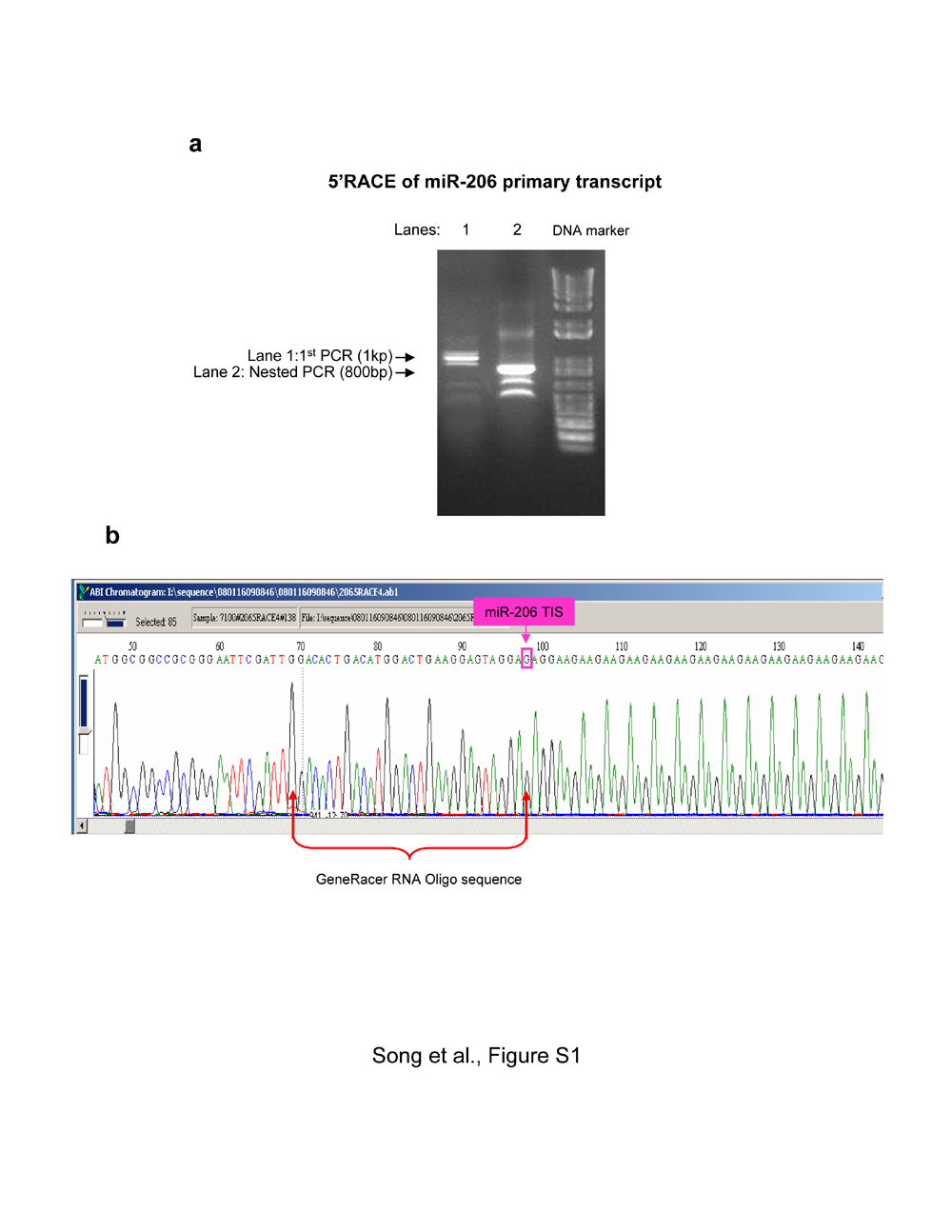

Supplement: Figure S1 — (a) Determining the putative transcriptional initiation site (TIS) by 5′-RACE for pri-miR-206. Total liver RNA was isolated using RNeasy Mini Kit (Qiagen, Valencia CA) and mRNA was isolated using a Oligotex Direct mRNA Mini Kit (Qiagen, Valencia CA). A GeneRacer Kit (Invitrogen, California USA) was used to map the transcriptional initiation site of the primary transcript. (b) Chromatogram of 5′ RACE sequences of the pri-miR-206 primary transcript. The putative transcriptional initiation site of pri-miR-206 (G) is indicated by a pink arrow. (3.89 MB TIF) [file pone.0006880.s003.tif]

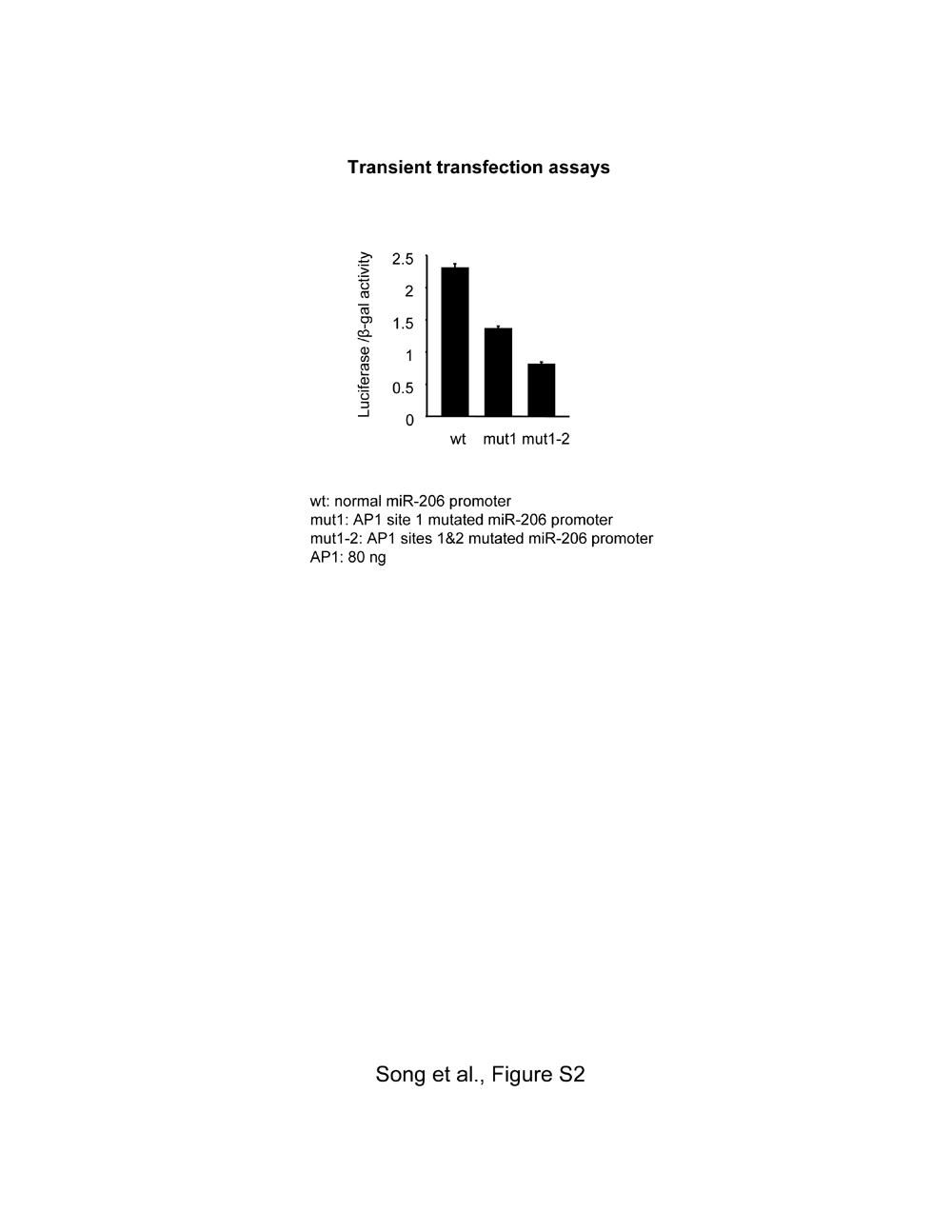

Supplement: Figure S2 — Mutagenesis assays. Two upstream putative AP1 sites (sites 1&2) (see Figure 2–1 for the location of each site) of the miR-206 promoter was mutated by using the QuickChange XL site-Directed Mutagenesis Kit (Stratagene), which generated the mutated miR-206 promoter luciferase reporter mut1 (single AP1 site 1 mutation) and mut1-2 (double AP1 sites 1&2 mutation). For luciferase reporter experiments, 30 ng of wt, mut1, mut1-2 and 30 ng of β-gal plasmid pSV-β-Galactosidase control vector were co-transfected with the AP1 expression vector (80 ng) into Hela cells using FuGENE HD (Roche). Thirty six hours after transfection, luciferase and β-galactosidase assays were performed using the Luciferase Assay System system and Beta-Glo® Assay System (Promega). Luciferase activities were normalized to galactosidase activities for each transfected well. For each experiment, wells were transfected in triplicate and each well was assayed in triplicate. (3.89 MB TIF) [file pone.0006880.s004.tif]

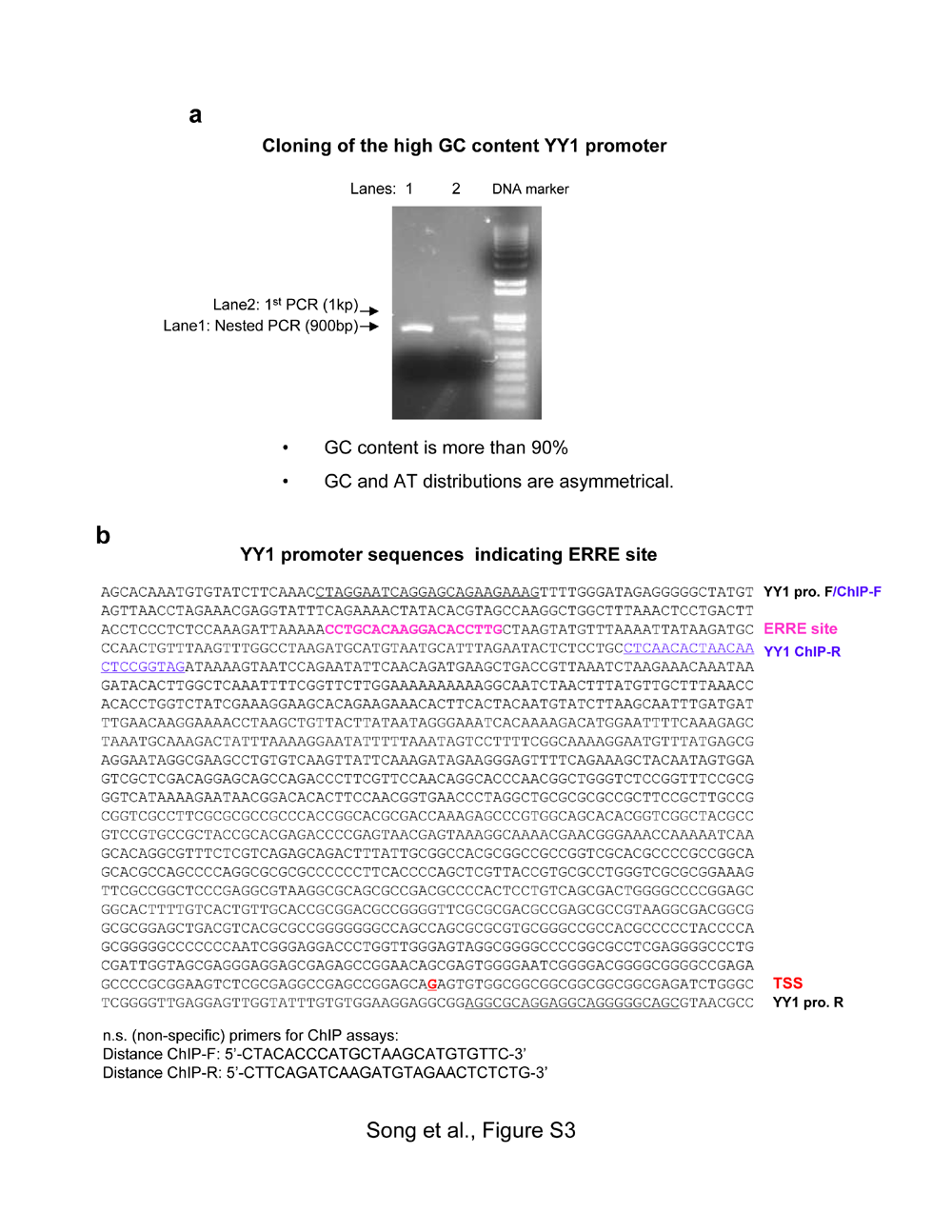

Supplement: Figure S3 — (a) Cloning of the high GC content mouse YY1 promoter. The PCR product was cloned into a pGL3-basic vector and used for transfection assays. The construct was verified by sequencing. (b) YY1 promoter sequences. TSS, transcriptional start site; ERRE, putative ERR binding site; YY1 pro. F and R, forward and reverse primers used to clone the YY1 promoter (pro.) and for ChIP assays. (3.89 MB TIF) [file pone.0006880.s005.tif]

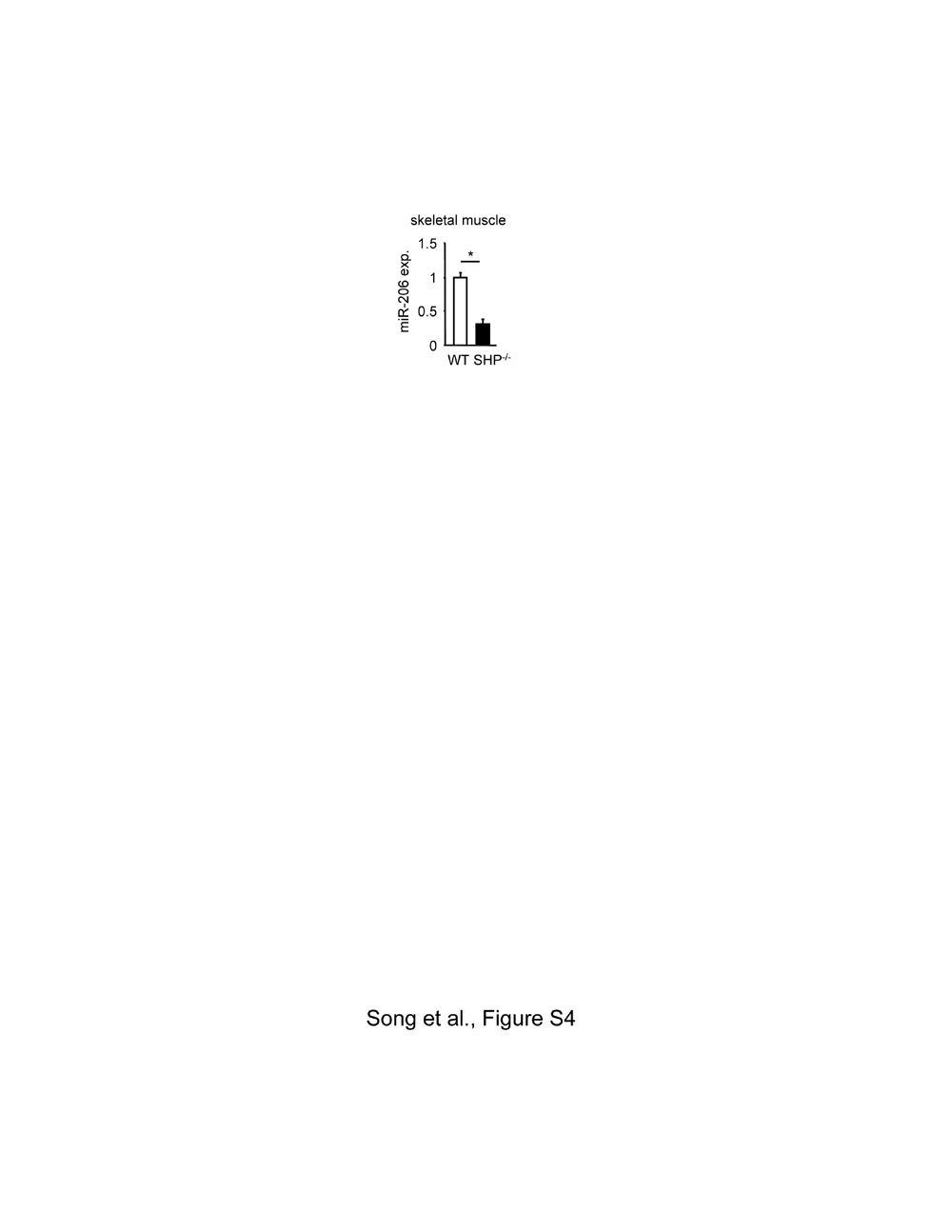

Supplement: Figure S4 — Real-time PCR analysis of miR-206 expression in skeletal muscle of SHP−/− and SHP+/+ mice. Data is represented as mean±SEM. *Significantly different (p<0.01). (3.89 MB TIF) [file pone.0006880.s006.tif]
